# Efficient Markerless Motion Classification Using Radar

**DOI:** 10.3390/s25113293

**Published:** 2025-05-23

**Authors:** Changhyeon Eom, Sooji Han, Sabin Chun, Soyoung Joo, Jisu Yoon, Min Kim, Jongchul Park, Sanghong Park

**Affiliations:** 1 Department of Physical Education, Graduate School, Pukyong National University, Busan 48513, Republic of Korea; ckdgus9902@naver.com (C.E.); 1004sabin0534@gmail.com (S.C.); jsy4560@naver.com (S.J.); julie7159@naver.com (J.Y.); 2 Industry-Academia Cooperation Foundation, Pukyong National University, Busan 48513, Republic of Korea; soojihan823@gmail.com; 3 Department of Maritime ICT & Mobility Research, Korea Institute of Ocean Science & Technology, 385, Haeyang-ro, Yeongdo-gu, Busan 49111, Republic of Korea; skymin@kiost.ac.kr; 4 Department of Marine Sports, Pukyong National University, Busan 48513, Republic of Korea; 5 Department of Electronic Engineering, Pukyong National University, Busan 48513, Republic of Korea

**Keywords:** 3-dimensional marker position, PCA, micro-Doppler, human body

## Abstract

**Highlights:**

**What are the main findings?**

- A novel markerless motion-classification method that uses radar micro-Doppler features achieves nearly 100% classification accuracy with a compact feature set.
- The method uses cross-correlation between radar signals and basis functions derived from 3D motion-capture data, then applies PCA dimensionality reduction and nearest-neighbor classification.

**What is the implication of the main finding?**

- The proposed method enables accurate classification of human motion without markers, and thereby ensures privacy and reduces the cost compared to traditional motion-capture systems.
- The approach is accurate across varying SNR conditions, and is therefore suitable for real-world applications such as healthcare monitoring and sports analytics.

**Abstract:**

This study proposes a novel method that uses radar for markerless motion classification by using effective features derived from micro-Doppler signals. The training phase uses three-dimensional marker coordinates captured by a motion-capture system to construct basis functions, which enable modeling of micro-motions of the human body. During the testing phase, motion classification is performed without markers, relying solely on radar signals. The feature vectors are generated by applying cross-correlation between the received radar signal and the basis functions, then compressed using principal component analysis, and classified using a simple nearest-neighbor algorithm. The proposed method achieves nearly 100% classification accuracy with a compact feature set and is accurate even at high signal-to-noise ratios. Experimental results demonstrate that to optimize training data and increase computational efficiency, the sampling duration and sampling interval must be set appropriately.

## 1. Introduction

Motion-capture systems for humans [1] are used in various domains, including healthcare [2] and sports [3]. Typically, motion has been captured using three-dimensional (3D) camera systems that track the coordinates of markers placed on the body [4]. However, markers interfere with natural movement, and their placement is often constrained [5]. Consequently, markerless motion capture is being evaluated. Nevertheless, they still use 3D camera systems, which are expensive and bulky [6]. Use of radar systems has been suggested as a method to overcome this problem [7].

Radar signals that reflect from targets can be measured without markers. Radar systems are significantly more affordable (a few hundred dollars) than 3D camera systems (tens of thousands of dollars). Additionally, radar’s compact size allows for flexible installation, whereas 3D motion-capture systems use fixed cameras and are therefore not easily portable. Furthermore, radar signals are less susceptible than 3D cameras to interference from optical signals, so radar systems can collect and process data faster than 3D camera systems. Radar can also observe under various conditions of weather and illumination, and by using wideband signals, can provide high-resolution data [8,9].

Accurate classification and analysis of human motion require precise modeling of micro-motions (MMs) of the human body, such as vibrations and rotations, with features derived from modeled motions serving as the basis. However, a human body is composed of several primary scatterers, each with a distinct location, speed of movement, and angular range of movement, and these traits vary significantly among individuals. Additionally, the MMs of each scatterer consists of multiple sine and cosine components with varying amplitudes, rotational velocities, and initial phases, so mathematical modeling of arbitrary MMs can be complicated. Therefore, analysis of electromagnetic data to model the MMs of the human body has not been sufficiently accurate.

The micro-Doppler (MD) effect [10] in radar technology refers to time-varying Doppler frequencies that are generated by MMs, and that represent these motions in the time–frequency domain. Analysis of the MD effect has clear applications to motion analysis. When radar observes a person performing specific movements, the motions of body parts such as the arms and legs produce corresponding MD signals. These signals are highly effective for motion recognition [11]. The radar spectrogram is a motion-analysis technique that depicts the power distribution of target returns in both the time and frequency domains [12].

Various pose-recognition techniques have been proposed; they primarily use neural networks to analyze spectrograms. Some of these classifiers can operate accurately using only limited training data [13,14,15]. To increase classification accuracy, data-augmentation methods can be used to expand training datasets [16,17,18], and transfer-learning approaches have also been explored [19,20,21,22]. However, methods that use neural networks can be accurate for predefined movements, but as the number of actions increases, the training phase requires substantial computational resources and time, and error rates often rise, so such approaches are not practical for use by simple systems.

Several methods that use empirical features in the time–frequency domain for specific MMs have been proposed for application to arbitrary motions, and in a limited capacity [23,24]. Techniques that derive the micro-Doppler image envelope of movements and compress it using the principal component analysis (PCA) for classification [25] are prone to noise interference and often struggle with envelope detection. For use when signal interference occurs, a method that uses empirical mode decomposition (EMD) to remove interfering signals has been proposed [26], but the method can yield inaccurate results if EMD fails to correctly represent the micro-Doppler features. Furthermore, the subsequent classification step requires a complex neural network structure.

This paper proposes an efficient method that uses ‘effective features’ to classify individual motions by using 3D marker coordinates obtained by a camera system only during the training phase, then classifies test data without the need for markers. The proposed features are derived from the MMs of the human body, and enable accurate classification using a minimal number of features. In experiments conducted using a continuous wave (CW) radar operating at 5.8 GHz, the proposed method achieved nearly 100% classification accuracy with a highly compact feature set and a simple classifier, and thereby demonstrates efficiency and effectiveness. The remainder of this paper is organized as follows. Section 2 introduces the signal model and the proposed method. Section 3 outlines the experimental conditions, while Section 4 presents the classification results and performance analysis. Finally, Section 5 concludes the paper and discusses potential directions for future work.

## 2. Signal Modeling and Proposed Method

### 2.1. Overview

The method constructs basis functions for MD features by using marker coordinates, and generates feature vectors by analyzing cross-correlation between the received radar signal and the basis functions. These feature vectors are then compressed using principal components analysis (PCA), and the reduced feature set is classified using a simple nearest-neighbor classifier. This approach eliminates the need for empirical analysis of motion because the basis functions are constructed using accurate marker data, so the features can clearly represent specific body parts.

### 2.2. Signal Modeling and the Proposed Feature

We assume a CW radar that continuously transmits and receives unmodulated carrier signals during the entire detection process. Although frequency-modulated CW (FMCW) radar offers additional information such as target range and angle, along with an increased signal-to-clutter ratio, we did not consider FMCW in this paper, because our primary focus is on developing an efficient recognition method that utilizes the frequency modulation induced by the body’s micro-motions. The radar signal transmitted at time *t* with a frequency *f*_0_ is expressed as

(1) $$s\left(t\right)=exp\left(j2\pi f_{0}t\right),$$

where $j=\sqrt{-1}$. When the transmitted signal is reflected from a target located at *r*(*t*), the received signal *s_r_*(*t*) is delayed as

(2) $$s_{r}\left(t\right)=exp\left(j2\pi f_{0}\left(t-t_{d}\right)\right)=exp\left(j2\pi f_{0}\left(t-\frac{2r\left(t\right)}{c}\right)\right),$$

where *c* = 3 × 10^8^ m/s represents the speed of light in a vacuum. The received signal *S_r_*(*t*) is down-converted to the baseband as

(3) $$s_{b}\left(t\right)=s_{r}\left(t\right)s\left(t{)}^{*}=\exp\left(j2\pi f_{0}\left(t-t_{d}\right)\right)\exp\left(-2\pi jf_{0}t\right)=\exp(-j2\pi f_{0}t_{d}\right)=\exp\left(-j\frac{2\pi }{\lambda }r\left(t\right)\right),$$

where *λ* = *c/f*_0_ is the wavelength.

When a target consists of *N_c_* scatterers, the received baseband signal is the sum of the reflected signals:

(4) $$s_{T}(t)=\sum_{i=1}^{N_{c}}s_{i}(t)=\sum_{i=1}^{N_{c}}A_{i}\exp\left(-j\frac{4\pi r_{i}(t)}{\lambda }\right),$$

where *A_i_* is the amplitude of scatterer *i*, and *r_i_*(*t*) is its distance. After the translational motion of the body is removed, *r_i_*(*t*) represents the MMs and can be expressed as

(5) $$r_{i}(t)=\sum_{j=1}^{N_{i}^{c}}\alpha_{i,j}\cos(\omega_{i,j}^{c}t+\phi_{i,j}^{c})+\sum_{j=1}^{N_{i}^{s}}\beta_{i,j}\sin(\omega_{i,j}^{s}t+\phi_{i,j}^{s}),$$

where $N_{i}^{c}$ represents the number of cosine components in scatterer *i*, which have amplitude $\alpha_{i,j}$, rotational velocity $\omega_{i,j}^{c}$, and initial phase $\phi_{i,j}^{c}$; and $N_{i}^{s}$ represents the number of sine components in scatterer *i*,, which have amplitude $\beta_{i,j}$, rotational velocity $\omega_{i,j}^{s}$, and initial phase $\phi_{i,j}^{s}$.

After *A_i_* of the target is fully extracted for 1 ≤ *i* ≤ *N_c_*, the MMs can be effectively recognized using a 1 × *N_c_* feature vector and a simple classifier. For a scatterer *k*, *A_k_* can be obtained by calculating a simple inner product between *s*(*t*) and a basis signal that eliminates the exponential component of *s_k_*(*t*) in (4). Therefore, assuming that *r_k_*(*t*) of a scatter *k* is completely known, the basis function to extract *A_k_* can be modeled as

(6) $$b_{k}(t)=\exp\left(-j\frac{4\pi r_{k}(t)}{\lambda }\right),$$

and *A_k_* can be obtained by using the inner product between *s*(*t*) and *b_k_*(*t*), which is

(7) $$\left\langle s_{T}(t),b_{k}(t)\right\rangle =\frac{1}{T}\int_{0}^{T}s(t)\exp\left(j\frac{4\pi r_{k}(t)}{\lambda }\right)dt\;=\frac{A_{k}}{T}\int_{0}^{T}1dt+\frac{1}{T}\sum_{i=1,i\neq k}^{N_{c}}A_{i}\int_{0}^{T}\exp\left(j\frac{4\pi \left(r_{k}(t)-r_{i}(t)\right)}{\lambda }\right)dt=A_{k}+R_{s}(t),$$

where *T* is the observation duration, and *R_s_*(*t*) represents the inner product between the remaining scatterers and scatterer *k*.

Using (5), *R_s_*(*t*) can be expressed as:

(8) $$R_{s}\left(t\right)=\frac{1}{T}\sum_{i=1,i\neq k}^{N_{c}}A_{i}\int_{0}^{T}\exp\left(\frac{4\pi j}{\lambda }\left(\begin{array}{c} \sum_{j=1}^{N_{i}^{c}}\left(\alpha_{i,j}\cos\left(\omega_{i,j}^{c}t+\phi_{i,j}^{c}\right)-\alpha_{k,j}\cos\left(\omega_{k,j}^{c}t+\phi_{k,j}^{c}\right)\right)\\ +\sum_{j=1}^{N_{i}^{s}}\left(\beta_{i,j}\sin\left(\omega_{i,j}^{s}t+\phi_{i,j}^{s}\right)-\beta_{k,j}\sin\left(\omega_{k,j}^{s}t+\phi_{k,j}^{s}\right)\right) \end{array}\right)\right)dt\;=\frac{1}{T}\sum_{i=1,i\neq k}^{N_{c}}A_{i}\int_{0}^{T}\exp\left(jM(t)\right)dt=\frac{1}{T}\sum_{i=1,i\neq k}^{N_{c}}A_{i}\int_{0}^{T}\left(\cos\left(M(t)\right)+j\sin\left(M(t)\right)\right)dt,$$

where

(9) $$M(t)=\left(\frac{4\pi }{\lambda }\right)\left(\sum_{j=1}^{N_{i}^{c}}\left(\alpha_{i,j}\cos\left(\omega_{i,j}^{c}t+\phi_{i,j}^{c}\right)-\alpha_{k,j}\cos\left(\omega_{k,j}^{c}t+\phi_{k,j}^{c}\right)\right)+\sum_{j=1}^{N_{i}^{s}}\left(\beta_{i,j}\sin\left(\omega_{i,j}^{s}t+\phi_{i,j}^{s}\right)-\beta_{k,j}\sin\left(\omega_{k,j}^{s}t+\phi_{k,j}^{s}\right)\right)\right).$$

*M*(*t*) is the phase of the complex exponential function, and obtaining the analytical integration of exp(*jM*(*t*)) is challenging because the parameters of the cosine and sine functions are generally unknown. However, for a periodic MMs and an integration duration *T* that is sufficiently longer than the period of the MM, then *M*(*t*) is also periodic. Consequently, the absolute value of the integration of exp(*jM*(*t*)) becomes much smaller than *T*, because the integral of a sinusoidal function over a complete period equals 0. Therefore, we can conclude that $\left|A_{k}\right|\gg \left|R_{s}\left(t\right)\right|$.

**Figure 1:**
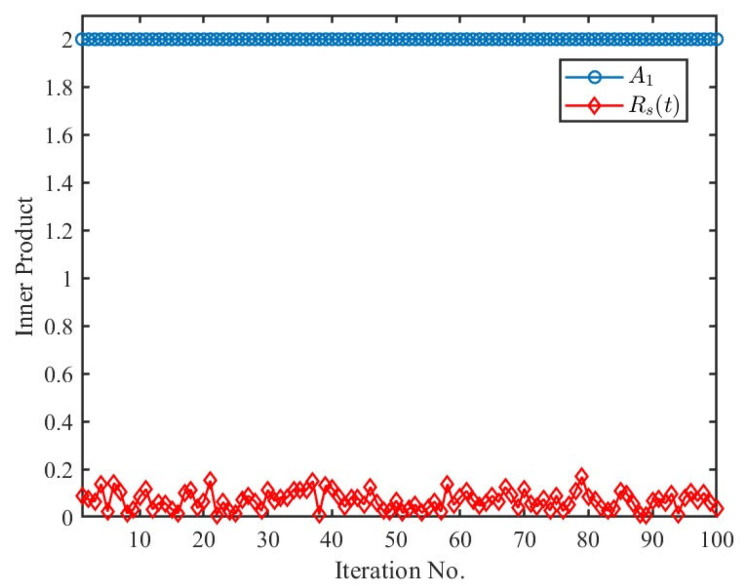
Comparison between *A_1_* and *R_s_*(*t*).

To demonstrate $\left|A_{k}\right|\gg \left|R_{s}\left(t\right)\right|$, a simulation was conducted. Assuming *f_0_* = 5.8 GHz, *T* = 3 s, sampling interval *dt* = 0.001 s, and four scatterers with equal amplitudes = 2, which have MMs that are each a sum of three cosine or sine components randomly selected, $\left|A_{1}\right|$ and $\left|Rs(t)\right|$ were compared 100 times using random MMs parameters. The frequencies were chosen randomly between 0 and 2 Hz, the amplitudes between 0 and 4π/*λ*, and the initial phases between 0 and 2π. All cases yielded $\left|A_{1}\right|$ in (8) equal to 2, and $\left|R_{s}\left(t\right)\right|$ remained close to 0 due to the periodic nature of the sinusoidal function (Figure 1).

Therefore, considering the difference in the initial phase, *A_k_* can be determined by finding the maximum inner product as

(10) $$A_{k}=\max\left\{\left\langle s(t),CS\left(b_{k}(t),\tau \right)\right\rangle \right\},\;\mathrm{for}\;0\leq \tau \leq T,$$

where $CS\left(b_{k}(t),\tau \right)$ is the signal of *b_k_*(*t*), after a circular shift by *τ*.

*A_k_* is found by identifying the maximum cross-correlation between *s*(*t*) and *b_k_*(*t*), as

(11) $$A_{k}=\max\left\{IFT\left\{FT\{s(t)\}FT\{b_{k}(t)\}\right\}\right\},$$

where IFT and FT represent, respectively, the inverse Fourier transform and the Fourier transform. In cases where the amplitude of scatterer *k* is small or a dominant scatterer exists, *A_k_* in (11) may not represent the exact amplitude of scatterer *k*. However, *A_k_* can still serve as an important feature because it quantifies the amount of MMs *r_k_*(*t*) that is present in *b_k_*(*t*).

### 2.3. Limitations in Determining Scatterer Amplitude and the Proposed Method

One- and two-dimensional methods that use spectral-estimation theory have been proposed to extract the amplitude and location of scatterers [27,28]. However, these methods assume that targets remain stationary while being observed, and are therefore unsuitable for estimating scatterers involved in MM. Additionally, selecting the dominant scatterer of the human body for classifying MMs is challenging, because the electromagnetic characteristics of individuals vary depending on the body’s composition (e.g., fat, muscle, bone). Although methods for modeling the human body have been suggested, they are often limited in their applications [29]. Moreover, modeling *r_k_*(t) becomes highly complex for arbitrary motions. Although some existing studies describe simple MMs such as walking or crawling [10], these approaches fail to capture the arbitrary motions of individuals. Therefore, extracting *A_k_* using (10) is a difficult task.

To address the limitations of existing methods, we propose utilizing marker data obtained by using a 3D motion-capture system only during the training phase. The use of a camera during the training phase does not invade privacy, because training datasets are generally constructed using individuals who have consented to being observed. This system, which is widely used for kinematic purposes, provides accurate 3D coordinates of body parts across sagittal, frontal, and transverse planes by using markers attached to the body, and more than two cameras. Therefore, employing this system effectively resolves the challenges in modeling MMs. The proposed approach builds on a previous simulation study [30] that demonstrated the feasibility of classification using marker coordinates and modeled radar signals with images in the time-frequency (TF) domain, despite the significant computational burden. This paper presents an improved method that eliminates the need for spectrograms, compresses the features further, and demonstrates effectiveness using measured data.

Using *N_c_* markers, *N_c_* basis functions can be derived using (6), and the following feature can be obtained using (11):

(12) $$f=[f_{1}f_{2}f_{3}\ldots f_{N_{c}}{]}^{T}.$$

For *P* motions, each with *Q* feature vectors, the following training database is constructed:

(13) $$F=[f_{1}f_{2}f_{3}\ldots f_{N_{tr}}]=\left[\begin{array}{cccc} f_{11} & f_{12} & \ldots & f_{1N_{tr}}\\ f_{21} & f_{22} & \ldots & f_{2N_{tr}}\\ \vdots & \vdots & \ldots & \vdots \\ f_{N_{c}1} & f_{N_{c}2} & \ldots & f_{N_{c}N_{tr}} \end{array}\right],$$

where *N_tr_* = *P* × *Q* represents the total number of training vectors, and *F* is an *N_c_* × *N_tr_* matrix.

Scatterers that generate large-magnitude signals may produce large elements in (11) and significantly influence the classification results, so each element should be normalized to values between zero and one by using the maximum and minimum values as [31]:

(14) $$\bar{F}=[{\bar{f}}_{1}{\bar{f}}_{2}{\bar{f}}_{3}\ldots {\bar{f}}_{N_{tr}}],$$

where

(15) $${\bar{f}}_{k}={\left[{\bar{f}}_{k1}{\bar{f}}_{k2}{\bar{f}}_{k3}...{\bar{f}}_{kN_{c}}\right]}^{T},$$

and

(16) $${\bar{f}}_{kj}=\frac{f_{kj}-f_{j,min}}{f_{j,max}-f_{j,min}}\;,\mathrm{for}\;1\leq j\leq N_{c}$$

where *f_j,min_* and *f_j,max_* are, respectively, the minimum and maximum values of the *j*-th feature in the training database.

In the test phase, only the radar signal is measured and used for classification, meaning the location for acquiring data is not constrained by the 3D motion system’s position. The feature vector is obtained using (12) with the measured radar signal, and the marker data *r_k_*(*t*) stored in the training database. The test vector is normalized using (16) to adjust the range of its elements.

### 2.4. Data Compression Using Principal Component Analysis and Classification Using a Simple Classifier

The exact number of scatterers in the human body cannot be precisely stated, so *N_c_* should be sufficiently large to encompass the MMs information of the entire body. However, setting *N_c_* excessively high can lead to redundant information, increased memory requirements for storing feature vectors, and excessive computational costs during classification, without increasing recognition accuracy. To address this problem, PCA was used [25]. This method is widely used for data compression while preserving discriminative information. The transformation matrix in PCA is designed to project the feature vector into a subspace spanned by the eigenvectors that correspond to the largest eigenvalues of the covariance matrix. This process extracts a representative of effective features while retaining most of the intrinsic information.

For PCA, the unbiased covariance matrix is calculated using the normalized feature vectors in the training database as

(17) $$Cov=\frac{1}{N_{tr}-1}\sum_{i=1}^{N_{tr}}\left({\bar{f}}_{i}-m\right){\left({\bar{f}}_{i}-m\right)}^{T},$$

where

(18) $$m=\frac{1}{N_{tr}-1}\sum_{i=1}^{N_{tr}}{\bar{f}}_{i}.$$

By the eigenvalue decomposition,

(19) $$Cov=V\Lambda V^{T},$$

where **V** is the matrix composed of the eigenvectors of **Cov** as its columns, and Λ is a diagonal matrix that contains the corresponding eigenvalues.

The transformation matrix **P** is then constructed by selecting the eigenvectors that correspond to the largest eigenvalues in descending order:

(20) $$P=\left[v_{1}v_{2}\cdots v_{d}\right],$$

where *d* is the number of largest eigenvalues, and **v**_1_–**v***_d_* are the eigenvectors that correspond to the largest *d* eigenvalues. $\bar{F}$ in (14) is then transformed by

(21) $$X=P^{T}\bar{F}=[x_{1}x_{2}x_{3}...x_{N_{tr}}],\;x_{k}=P^{T}v_{k},$$

where the resultant feature vector **x***_k_* is *d*-dimensional, i.e., significantly smaller than *N_c_*.

**x***_k_* provides distinctive features for each MM, so a simple nearest neighbor classifier is used. This classifier uses the Euclidean norm for classification:

(22) $$\hat{s}=\underset{s}{min}g_{s}\left(x_{te}\right),$$

with

$$g_{s}\left(x_{te}\right)=\left\|x_{te}-x_{s}\right\|,$$

where $x_{te}$ is a test vector, and $x_{s}$ is a training vector for the *s*-th motion. The observation includes *P* MMs, so $\hat{s}$ ranges from one to *P*.

### 2.5. Overall Procedure

**Figure 2:**
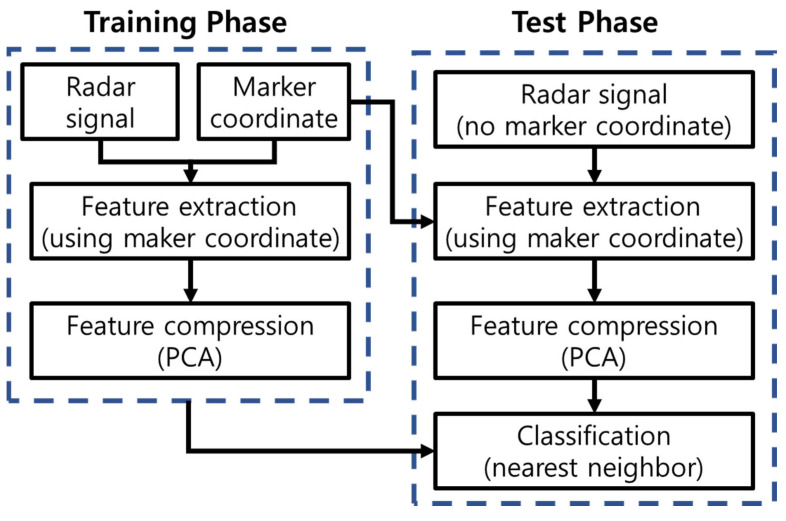
Overall classification procedure.

The overall procedure proposed in this paper is summarized as follows (Figure 2):

1. Data collection: For each of the *P* MMs, collect *Q* radar signals and *Q* × *N_c_* marker coordinates;
2. Signal construction: Using the marker coordinates, construct *bk*(*t*) as defined in (6);
3. Feature vector construction: For each radar signal, construct an *N_c_* × 1 feature vector using the maximum correlation, as described in (11) and formatted in (12);
4. Training database construction: Construct the training database F as defined in (13);
5. Normalization: Normalize the feature vector in F using the maximum and minimum feature values, as described in (15);
6. Compression: Compress the normalized feature vector using PCA;
7. Test motion processing: For any randomly selected test motion, repeat steps (1)–(3) and (5)–(6). In this step, the marker coordinates from the training database are used to construct the test feature; (11) is applied to the measured test signal;
8. Classification: Classify the motion using a simple nearest neighbor classifier.

## 3. Experimental Conditions

### 3.1. Participants

The experimental subjects were six adult males and four adult females (Table 1), none of whom had injured their upper or lower extremities in the previous six months. All participants were fully informed about the purpose of the research, experimental procedures, and precautions, and data collection was conducted only after obtaining their consent to participate. The experiment was approved by the Institutional Review Board (IRB # PKNU 2024-12-002) of the university.

### 3.2. Motions and Camera System to Extract Marker Information

**Figure 3:**
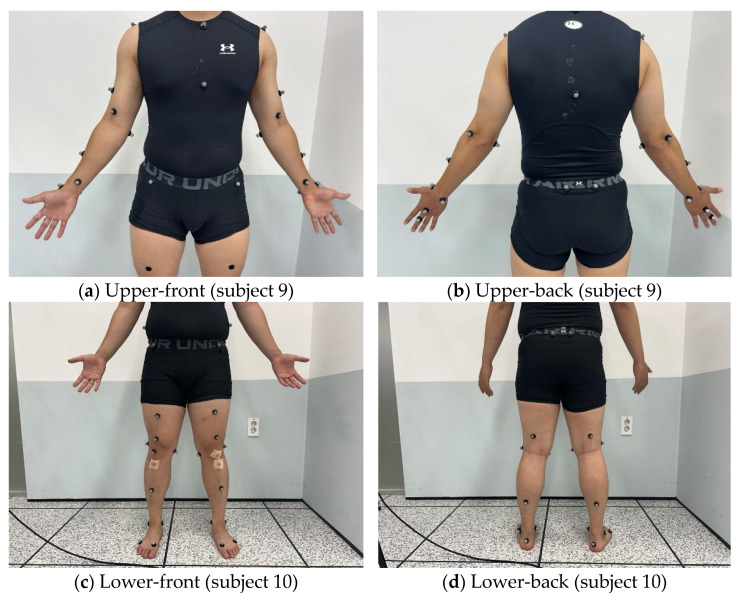
Marker positions.

Five representative motions were used for classification: squat, lunge, front kick, front lateral raise, and arm curl. To obtain position data for the markers, eight optical motion-capture cameras (Miqus M5, Qualisys AB, Gothenburg, Sweden) with a sampling rate of 250 Hz were used. Calibration was performed using the non-linear transformation method to define 3D spatial coordinates. Each participant had a total of 30 reflective markers (12 mm in size) attached to both the upper and lower body, particularly in areas where MMs occur frequently (Figure 3). Additionally, markers were attached to the back of the body to account for penetration of electromagnetic waves. The position data of the markers were obtained using Qualisys Track Manager; Qualisys, Gothenburg, Sweden software (QTM 2024.3), and the radar was modeled using MATLAB R2023b.

### 3.3. Radar and Classification Parameters

In the measurement, the radar signal and the marker coordinates were obtained simultaneously for *T_end_* = 4 s. A CW radar was used, positioned 1.5 ± 0.5 m along the y-axis from the subject. The distance between the radar and the body parts was determined using the markers attached to both the radar and the participant. The radar coordinate was set to (0, 0, 0) by subtracting the radar’s marker coordinates from the participant’s marker coordinates. The center frequency of the CW radar was 5.8 GHz, corresponding to a wavelength *λ* = 0.0517 m, and the sampling rate was set to 5 kHz (Table 2). For the experiments, we used MATLAB R2023 running on Windows 11 operated by AMD Ryzen 9 processor.

**Figure 4:**
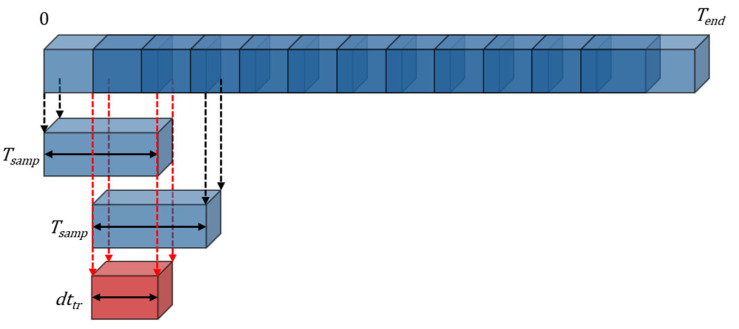
Construction of the training data sampled at uniform time interval.

Simultaneously, the camera system captured marker coordinates to construct the basis function (Equation (6)). To create the training dataset for each movement, the total time-domain signal of duration *T_end_* was segmented into signals of length *T_samp_* = 0.3 × *T_end_* = 1.2 s. These segments were clipped at uniform intervals of *dt_tr_* = 0.02 s, producing *N_tr_* = (*T_end_* − *T_samp_*)/*dt_tr_* = 140 training signals per participant (Figure 4). For each clipped signal, a feature vector was generated using (11). Each feature was normalized as per (16), and dimensionality reduction was performed using PCA, reducing the vector to *d* = 4 dimensions as specified in (21). Classification was conducted using 50 randomly clipped test signals per motion, each of the same length *T_samp_*. To evaluate the effect of various parameters on classification accuracy, additional experiments were performed at various signal-to-noise ratios (SNRs) by introducing additive white Gaussian noise (AWGN) to the test data (Table 2). The classification accuracy *P_c_* was defined as

(23) $$P_{c}=\frac{N_{cc}}{N_{s}}$$

where *N_cc_* is the number of the correct classifications and *N_s_* is that of the test samples.

## 4. Classification Results

### 4.1. Analysis of MMs for Each Motion

**Figure 5:**
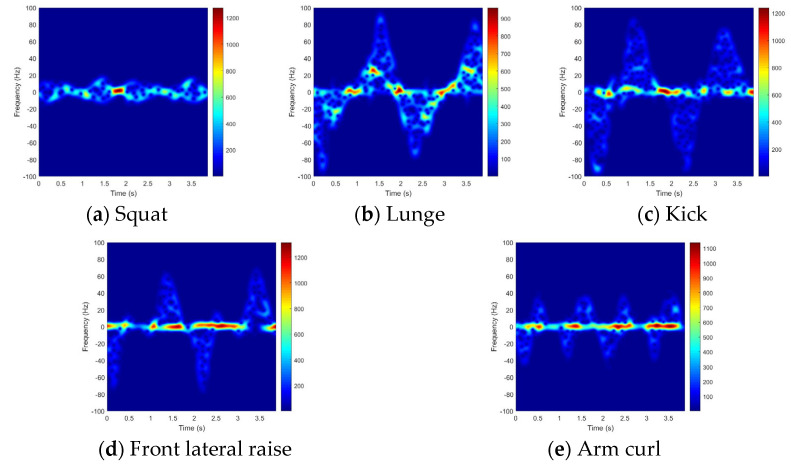
TF image of each motion (simulated).

**Figure 6:**
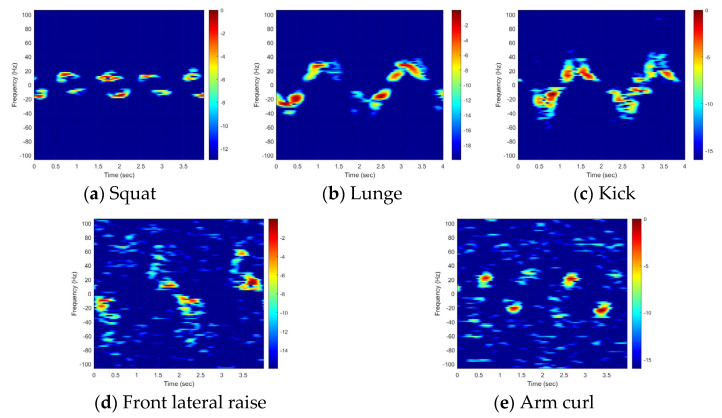
TF image of each motion (measured).

A comparison of the TF images derived from the simulated data using marker coordinates and those from the measured data revealed both similarities and differences (Figure 5 and Figure 6). Marker coordinates and radar signals were obtained simultaneously, so the time periods and Doppler-frequency magnitudes in the simulated data were strongly similar to those in the measured data. Consequently, the proposed feature in (11) produces high values when the modeled signal aligns closely with the measured one, and low values when a mismatch occurs.

The observed differences arise from the electromagnetic scattering characteristics. In the simulation, markers were assumed to be omnidirectional scatterers with consistent amplitude, and to remain visible throughout the simulation period and provide precise Doppler frequencies. However, in reality, the scatterer’s position is unknown and varies with the aspect angle, so the signal amplitude changes significantly during the observation period. As a result, (11) may yield suboptimal results when a particular motion produces weak or very strong scatterers.

**Figure 7:**
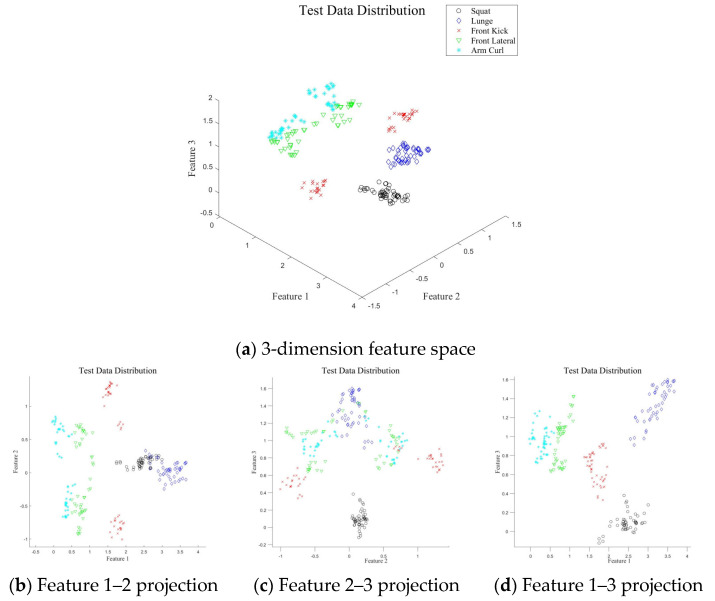
Feature vectors of each motion (*d* = 3).

The unique characteristics of each exercise were well-distinguished in a three-dimensional feature space (feature 1, feature 2, feature 3) when *d* = 3 (Figure 7). Features of front lateral raise and arm curl appear close or partially overlapping due to their reliance on arm movement alone, so the two movements have similar Doppler characteristics (Figure 7b–d). This overlap highlights the necessity for densely sampled training data to increase classification accuracy for these motions. On the feature 1–2 plane (Figure 7b), squat and lunge features are close, so they may be misclassified. However, in the feature 2–3 and 1–3 planes (Figure 7c,d), these motions are distinguishable due to their separation in these dimensions. Similarly, front kick features are not well-separated in the feature 2–3 plane, but are distinguishable in the feature 1–2 and 1–3 planes, in which their unique characteristics become evident. These observations suggest that use of the three-dimensional feature space has the potential to achieve high classification accuracy by using the distinct separation of features across different planes.

### 4.2. Classification Result for Various SNRs and Analysis on the Effect of PCA Dimensions

To evaluate classification accuracy in relation to the PCA dimensionality of training data and the SNR of test data, we assessed accuracy by varying PCA dimensions from 3 to 6 and SNR values from 0 dB to 20 dB in increments of 5 dB. The noise inherent in the measured data was not considered when adding AWGN, so the actual classification SNR was lower than the assumed SNR. Default radar and classification parameters (Table 2) were applied. Additionally, *P_c_* for each motion was analyzed in detail.

**Figure 8:**
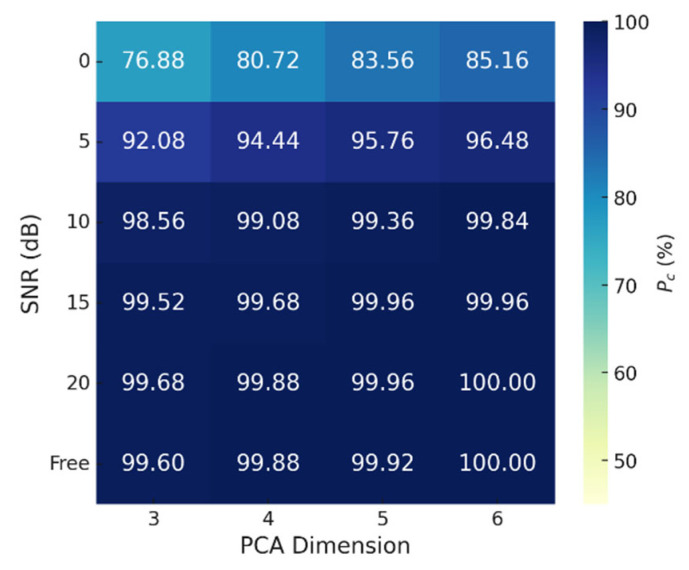
*P_c_*s of entire motions for various PCA dimensions (*d*) and SNRs.

**Figure 9:**
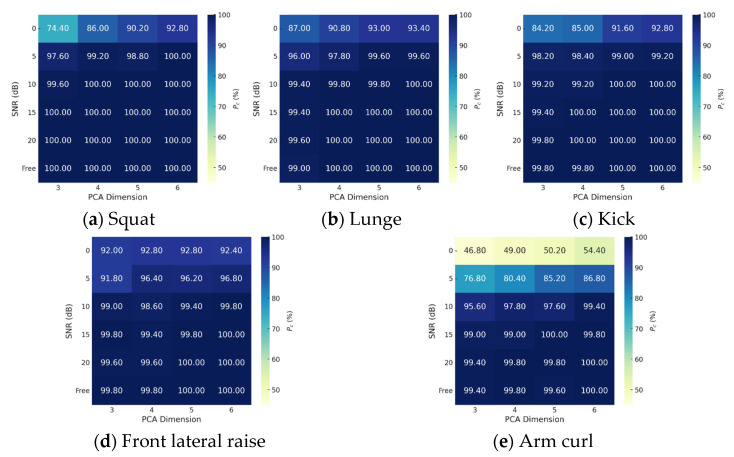
*P_c_*s of each motion for various PCA dimensions (*d*) and SNRs.

The proposed method demonstrated consistently high *P_c_*, even with low PCA dimensions, and increasing the dimensionality increased the accuracy. Similarly, higher SNR values corresponded to better classification accuracy (Figure 8 and Figure 9). Among the motions, the classification accuracy for arm curl was much lower for SNRs ≤ 5 dB than at other SNRs, due to its similarity to front lateral raise. The proximity of their features resulted in some arm curls being misclassified as front lateral raises (Figure 7b–d). Overall, the results indicate that achieving *P_c_*s > 99.0% requires an SNR ≥ 10 dB and *d* ≥ 4. Given the sample size of the measured data = 5 kHz (sample rate) × 1.2 s (*T_samp_*) = 6000, *d* = 4 corresponds to only 0.07% of the measured data. This result demonstrates the remarkable efficiency of the proposed feature-extraction method.

### 4.3. Analysis of the Classification Accuracy of Each Motion

**Figure 10:**
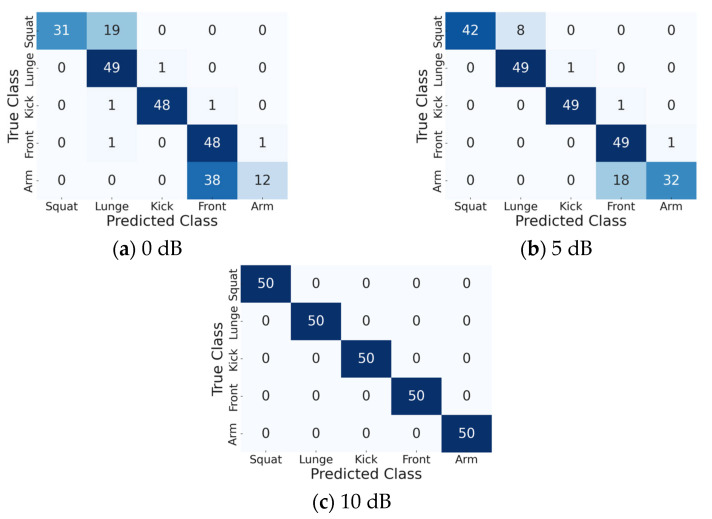
Confusion matrices for three SNRs.

The classification accuracy of each motion was analyzed using the confusion matrix obtained at SNRs of 0, 5, and 10 dB, with *d* = 6 as the default parameter (Figure 10). The classification accuracy improved proportionally with the SNR, with a classification rate of 100% achieved at an SNR of 10 dB. This demonstrates that an SNR of 10 dB or higher is required, as also observed in Figure 8. Notably, the front kick was correctly classified even at low SNR values, owing to its distinctiveness from the other motions. However, the squat–lunge and front lateral raise–arm curl pairs were misclassified due to the similarity in their MDs. Specifically, as seen in the TF image (Figure 6), the front lateral raise and arm curl motions were very similar, resulting in significant misclassification of the arm curl motion, with 38 out of 50 misclassifications at SNR = 0 dB and 18 out of 50 misclassifications at SNR = 5 dB. This result indicates that further research is necessary to enhance the accuracy of the proposed method at low SNRs.

### 4.4. Analysis of the Training Database

#### 4.4.1. Effect of *dt_tr_* in the Training Database

**Figure 11:**
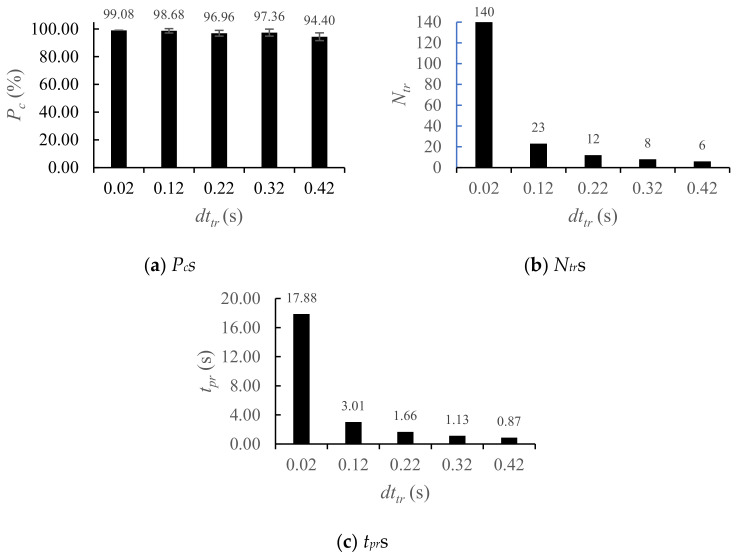
*P_c_*s, *N_tr_*s, and *t_pr_*s for various *dt_tr_*s.

Considering the results from Section 4.2, the effect of *dt_tr_* for training data on classification accuracy was analyzed with *d* = 4 at SNR = 10 dB. Using *T_samp_* = 1.20 s and *T_end_* = 4.00 s, classifications were performed with *dt_tr_* = 0.02 s to 0.42 s in increments of 0.10 s. The classification accuracy remained insensitive to changes in *dt_tr_*, despite a significant reduction in the number of training samples. Specifically, the number *N_tr_* of training vectors per target decreased from 140 to just 6 (Figure 11a,b). Although *N_tr_* was reduced to 4.29% of its original value, *P_c_* only declined by 4.68%; this result demonstrates the efficiency of the proposed feature extraction method. Notably, an accuracy of *P_c_* = 98.68% was achieved with *dt_tr_* = 0.12 s, meaning only 23 feature vectors of dimension 4 per motion were sufficient to produce results approaching 100% accuracy. Additionally, the processing time *t_pr_* for constructing the training data dropped by 95.13% from 17.88 s at *dt_tr_* = 0.02 s to just 0.87 s at *dt_tr_* = 0.42 s (Figure 11c). This result demonstrates the computational efficiency of the proposed approach.

#### 4.4.2. Effect of *T_samp_*

**Figure 12:**
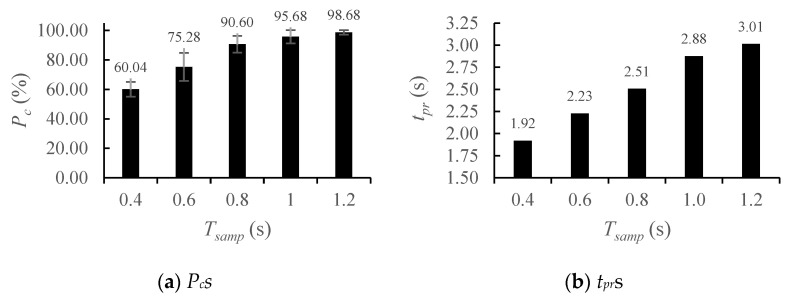
*P_c_*s and *t_pr_*s for various *T_samp_*s.

To analyze the effect of *T_samp_* of the TF on classification accuracy, we varied *T_samp_* from 1.2 s to 0.4 s in increments of 0.2 s, using SNR = 10 dB, *d* = 4 and *dt_tr_* = 0.12 s, as determined in the previous subsections. Classification accuracy was strongly dependent on *T_samp_*; this result emphasizes that accurate classification of MMs requires samples of sufficient duration (Figure 12a). Although reducing *T_samp_* shortened *t_pr_* (Figure 12b), the classification accuracy decreased due to insufficient time representation of MM. Therefore, because the MMs typically varies over time with a characteristic period, *T_samp_* must be sufficiently large to allow the features to clearly represent each MMs. In our experiment, MM periods exceeded 1.2 s, so *T_samp_* ≥ 1.2 s was necessary to achieve *P_c_* > 99% by ensuring distinct feature separation in feature space. Conversely, *t_pr_* increased as *T_samp_* increased, i.e., from 1.92 s at *T_samp_* = 0.4 s to 3.01 s at *T_samp_* = 1.2 s.

### 4.5. Limitations of the Proposed Method and the Solution

The proposed method assumes recognition of a single subject, so several challenges remain to be addressed. In this subsection, we analyze key factors that must be overcome to decrease the sensitivity of the model to variations in conditions.

**Figure 13:**
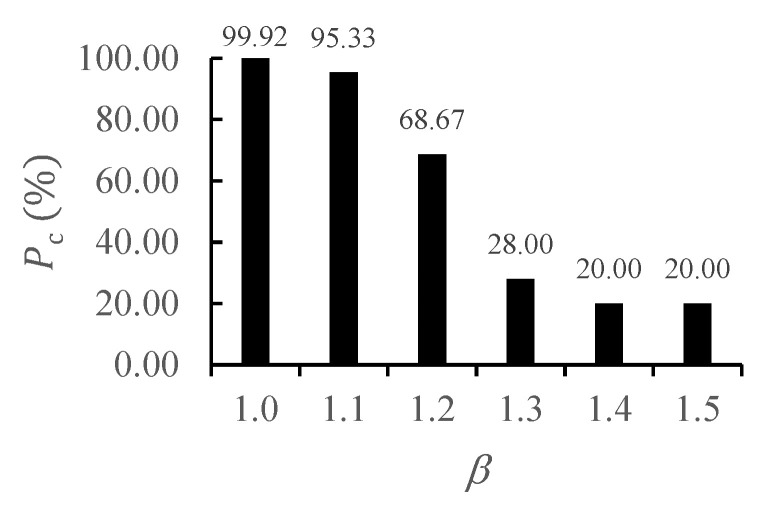
*P_c_*s for time-scaling.

The first challenge concerns inter-individual variability in movement speed. In our experiments, the training and test data had similar speed characteristics, so *P_c_*s were close to 100%. However, significant differences in movement speed between individuals can lead to substantial mismatches between training and test data. To examine the impact of this variation, we temporally scaled the test data in time-domain by a factor *β*, ranging from 1.0 to 2.0 in increments of 0.1, under an SNR of 30 dB. As *β* increased, *P_c_* dropped sharply; this result indicates that the current model is sensitive to variation in the speed of movement (Figure 13). This result underscores the need to either widen the range of speed profiles in the training data or develop an effective rescaling mechanism to adapt to individual differences in movement speed.

The second challenge lies in addressing scenarios in which multiple subjects perform various motions. The proposed method was originally designed without accounting for multiple objects, so applying its features to test vectors composed of random combinations of motions can result in significantly degraded tacking accuracy. To evaluate the effect of multiple subjects, we randomly selected and summed two test data samples at SNR = 30 dB, then applied the proposed method for classification. The resulting *P_c_* was 21.2% (Table 3); this low accuracy emphasizes the need for a dedicated feature representation that can distinguish concurrent motions of multiple entities. This accuracy degradation may be mitigated by adopting a new protocol similar to one that was recently developed for recognizing multiple drones or jets [32,33], which constructs the training dataset by combining instances from single-target training data.

The third challenge concerns the classification of diverse populations using a limited training dataset, particularly when accounting for variations in body types, age groups, or mobility impairments. To highlight this problem, we performed a classification experiment at SNR = 30 dB, in which 50% of the test signals in the time domain were randomly selected and their amplitudes and phases were independently varied within ±50%. As in the case of multiple motion classification, the resulting *P_c_* was degraded (77.3%); this result indicates the need for a fundamental analysis of individual RCS variability in relation to factors such as gender, body size, and other anthropometric differences (Table 3).

Moreover, real-world factors such as posture changes and non-Lambertian RCS variations can introduce significant classification errors. These factors must either be explicitly accounted for or addressed by the development of robust algorithms. Posture changes, for instance, can be treated relatively simply as anomalies during recognition, or incorporated into the training process using the proposed method. In contrast, RCS variation in humans is a particularly complex problem, because it depends heavily on individual body composition. Accurate modeling of such variations requires extensive experimentation that considers a wide range of influencing parameters including aspect angle, operating frequency, environmental conditions, and even the amount of perspiration on the skin.

## 5. Conclusions

This paper has presented a novel method for motion classification using training data derived from markers and radar signals. Results demonstrated the feasibility of achieving high accuracy without relying on markers in the testing phase. By using PCA for dimensionality reduction and a nearest-neighbor classifier, the method achieved excellent classification accuracy even with compact feature sets, and at various SNRs. In particular, the use of 3D marker coordinates during the training phase enabled reliable feature representation, allowing effective classification of radar signals in the testing phase without markers. The results confirm that the use of data obtained using markers can serve as an effective foundation for motion analysis radar, and that this use delivers high accuracy and efficiency. In our experiments, the parameters that achieved *P_c_* ≥ 99.8% were *d* = 6, *dt_tr_* = 0.02 s, and *T_samp_* = 1.2 s at SNR = 10 dB. However, classification accuracy was highly sensitive to these parameters, and therefore, these settings must be optimized for other motions.

However, this study utilized training and testing datasets derived from only ten subjects to verify classification accuracy. Future research should involve testing with datasets generated from additional subjects to evaluate the generalization and reliability of the radar-based motion recognition algorithm across diverse individuals. Additionally, expanding the radar’s field of view and integrating additional data sources could increase the precision of motion-recognition algorithms. This study provides a strong foundation for radar-based motion analysis technologies, by demonstrating their potential for high accuracy and efficiency in various fields such as healthcare, sports analytics, and security.

## Figures and Tables

**Table 1:** Characteristics of subjects (mean ± s.d).

Variables (Mean ± Std)	6 Males	4 Females
Height (cm)	174.00 ± 5.33	166.38 ± 3.82
Weight (kg)	82.00 ± 11.03	57.50 ± 6.45
Age (y)	25.67 ± 1.21	24.50 ± 2.38

**Table 2:** Parameters for experiment.

Parameter	Value	Parameter	Value
*f_c_*	5.80 GHz	*λ*	0.05 m
*T_end_*	4.00 s	*T_samp_*	1.20 s
*dt_tr_*	0.02 s	*N_tr_*	140
*d*	4	SNR	0–20 dB

**Table 3:** Result of classification for two motions and body variation.

Single Motion	Two Motions	Body Variation
99.12%	21. 2%	77.3%

## Data Availability

The original contributions presented in this study are included in the article. Further inquiries can be directed to the corresponding authors.
